# iRhom2 in the pathogenesis of oral squamous cell carcinoma

**DOI:** 10.1007/s11033-020-05381-y

**Published:** 2020-03-31

**Authors:** Matthew E. Agwae, Richard J. Shaw, Asterios Triantafyllou, Frances S. T. Greaney, Khaled Ben Salah, Janet M. Risk

**Affiliations:** 1 Cancer Research Centre, Department of Molecular and Clinical Cancer Medicine, Institute of Translational Medicine, University of Liverpool, 200 London Road, Liverpool, L3 9TA UK; 2 Department of Oral & Maxillofacial/Head and Neck Surgery, Aintree University Hospital, NHS Foundation Trust, Liverpool, L9 7AL UK; 3 Department of Pathology, Liverpool Clinical Laboratories, University of Liverpool, Liverpool, UK

**Keywords:** iRhom2, Oral squamous cell carcinoma, Head and neck squamous cell carcinoma, Cell migration

## Abstract

iRhom2 is an inactive rhomboid protease involved in diverse signalling events. It has been implicated in the pathogenesis of a number of cancer types, including oesophageal and ovarian cancer, while its closely associated family member, iRhom1, is implicated in head and neck cancer. However, a role for iRhom2 in head and neck cancer has not been investigated. Immunoblotting for iRhom2 in 54 oral squamous cell carcinoma (OSCC) and 24 paired normal tissues demonstrated higher levels of iRhom2 protein in tumour compared with normal samples (P < 0.05). iRhom2 over-expression correlated with poor patient survival (P < 0.0005) but with no other clinicopathological variable. Increased cell migration was observed in stably over-expressing iRhom2 clones of OSCC cell lines in the absence of increased cell proliferation, but not in the normal oral keratinocyte cell line, NOK-hTERT, and this was abrogated by knock-down of iRhom2. iRhom2 protein expression is increased in a proportion of OSCC and this up-regulation is associated with faster cell migration and decreased patient survival. These data implicate iRhom2-controlled signalling events in the pathogenesis of this cancer.

**Electronic supplementary material:**

The online version of this article (10.1007/s11033-020-05381-y) contains supplementary material, which is available to authorized users.

## Introduction

Rhomboids are intramembrane serine proteases, with their active and catalytic residues located within the cell membrane lipid bilayer, enabling them to easily cleave and activate / inactivate membrane proteins within their transmembrane helices [1]. Although lacking protease function, iRhom2 has been shown to be involved in diverse signalling events such as the trafficking/activation of important growth and signalling factors, e.g. ADAM17, EGFR, and TGFα [2, 3]. iRhom2 has been implicated in the pathogenesis of a number of cancer types including oesophageal and ovarian cancer [4], while its closely associated family member, iRhom1, is implicated in EGFR signalling in head and neck cancer [5], but evidence for a specific role for iRhom2 in head and neck squamous cell carcinoma (HNSCC) has not been described.

HNSCC was reported to be the eighth most common cancer type in the UK in 2014. Worldwide, it is the sixth leading cancer type by incidence, with more than 550,000 cases and about 300,000 deaths in 2014 [6]. The 5 year overall survival in this cancer remains at approx. 50% [7] despite advances in surgical and chemoradiation treatments, implicating hitherto unexplored pathways..

This investigation focuses on the possible oncogenic role of iRhom2 in oral squamous cell carcinomas, the most common form of HNSCC, and its possible utility as a biomarker for survival.

## Materials and Methods

### Patients

Fresh, frozen tissues were obtained from 54 OSCC tumours and 24 paired normal adjacent tissues. Written consent was obtained from all patients (REC numbers: EC.47.01 & 10/H1002/53). The tissues were snap frozen at point of surgery and stored at -80°C prior to use. Demographic and clinicopathological data was collected.

Frozen tissues were embedded in OCT and a 5 μm section assessed for tumour presence. Tumour samples with less than 60% tumour content were excluded. Normal samples with more than 5% tumour content were also excluded.

### Cell lines and cell culture

The oral cancer cell lines, PE/CA-PJ15 [8] (source: ECACC) and Liv37K, a locally derived, low passage cell line, both showed detectable levels of iRhom2 expression and were selected to represent an established oral cancer cell model and a ‘close to the tumour’ oral cancer cell model, respectively. The normal oral keratinocyte cell line, NOK-hTERT [9] (Source: Prof Karl Münger, The Channing Laboratory, Brigham and Womens Hosptial, Boston, USA) was selected as a control. All cell lines were cultured at 37 °C in 5.0% carbon dioxide. Liv37K was used at or below passage 10 for all experiments reported here.

Cell monolayers were scraped into 10 ml PBS, followed by centrifugation to recover cell pellets which were stored at − 80 °C prior to protein extraction. The identity of the cell lines was confirmed by STR typing using GenePrint10® (Promega) and were mycoplasma negative by E-Myco PCR detection kit (CHEMBIO Ltd).

### Protein extraction

5 mm^3^ pieces of tissue were excised from areas with an increased number of tumour cells or lack of tumour as appropriate (determined from H&E sections) and re-suspended in protein extraction buffer (50 mM Tris–HCl, pH 6.8, 1% SDS, 1% EDTA, 0.25% glycerol, 0.25% β-mercaptoethanol with protease inhibitor (Roche Diagnostics), and sonicated for 30 s to extract total protein which was stored at − 80 °C until use. Recovered cell pellets were re-suspended in protein extraction buffer and similarly processed.

### Western blot analysis

Protein samples were loaded unto 10% SDS/PAGE gels. TE3-RHBDF2, an oesophageal cancer cell line constitutively expressing iRhom2, was used as a positive control. Following electrophoresis, gels were transferred to 0.45 μm PVDF membranes (GE Healthcare Life Sciences) and western blotting carried out. Membranes were blocked for one hour in PBS/Licor Blocking Buffer, ratio 1:1 then probed, using rabbit polyclonal anti-RHBDF2 (1:500: Sigma Aldrich: HPA018080) or mouse monoclonal anti-β-actin (1:1000: SantaCruz: sc-4778) at room temperature for 2 h. Fluorescently labelled secondary antibodies; IRDye@800 goat anti-rabbit (Licor) and Alexa Flour@680 rabbit anti-mouse (Life Technologies), were used at a concentration of 1: 10,000. Visualisation of fluorescent secondary antibody localisation was undertaken on a Licor Odyssey 3.0 imager (Licor Biosciences UK Ltd).

Densitometry was used to normalise and semi-quantitate the levels of protein expression in comparison with the actin control and the positive control sample run on each gel. Samples were ascribed a status of high (or overexpression) or moderate (or normal expression) based on a cut-off of 19.9 relative absorbance units, which was determined to be the boundary of the 75^th^ percentile from the cumulative frequency curve of the expressing samples**.** Samples demonstrating no measurable expression were classified as no expression.

### Stable transfection

Clones of PE/CA-PJ15, Liv37K and NOK-hTERT that stably over-expressed *RHBDF2* were created by transfection with a 5.3 kb pIRESneo vector (Clontech) containing isoform 2 of *RHBDF2* (Epoch Life Science Inc)*.* shRNA knock-down of *RHBDF2* gene was achieved using Mission®shRNA (SIGMA—09051604MN) with Mission®pLKO.1-puro B2M shRNA control (SIGMA-SHC008-04021321MN)( Sigma-Aldrich/Merck).

### Proliferation assay

A crystal violet assay was used to assess proliferation. Briefly, cells were seeded into 6 well plates and harvested daily for 5 days, then stained with 0.05% crystal violet solubilised in 10% acetic acid and absorbance read at 570 nm using a Spectramax-Plus284 absorbance microplate reader (Molecular Devices) against a 10% acetic acid blank.

### Migration assay

Cells were seeded in triplicate into removable chambers (Ibidi, Thistle Scientific) placed in 6 well plates, then cultured until they reached approximately 85% confluency. The removable inserts were withdrawn and the plates transferred to culture chamber attached to a Nikon Eclipse TE300 microscope for image capture at ten minutes intervals for 30 h. Gap closure images were analysed on Image J (https://imagej.nih.gov/ij/) and T-Scratch [10].

### Statistical analysis

Protein expression data from tissue was analysed using box plot analysis and paired sample t test (SPSS statistics, version 24). Chi-squared and the Kaplan–Meier survival tests were used to analyse associations between tissue expression and clinicopathological features.

## Results

### Expression of iRhom2 in OSCC and normal oral tissues

**Fig. 1:**
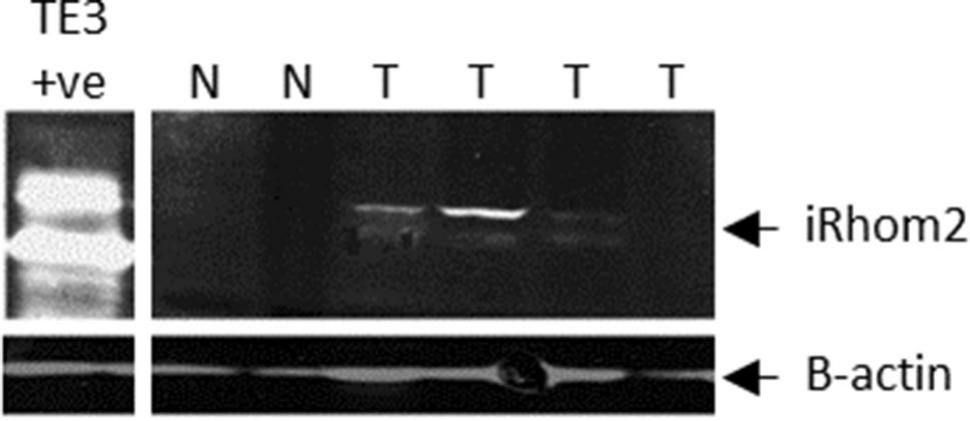
Representative western blot of iRhom2 expression in normal and tumour tissue. *N* normal tissue, *T* tumour tissue, *TE3 + ve* cell line overexpressing iRhom2 (+ ve control for normalisation between gels)

iRhom2 protein was highly expressed in 19/54 (35%) tumour samples, moderately expressed in 17/54 (31%) tumour samples, and undetected in the remaining 18/54 (33%) (Fig. 1, Table 1). In contrast, iRhom2 protein was highly expressed in only 1/24 (4%) normal samples, moderately expressed in 5/24 (21%) normal samples and undetectable in the remaining 18/24 (75%) (P < 0.05).

**Table 1:** Distribution of levels of iRhom2 protein expression in oral squamous cell tumour and adjacent normal tissue determined by western blotting

iRhom2 protein expression^a^	Tumour tissue (n = 54)	Normal tissue (n = 24)
High (> 19.9 arbitrary densitometry units)	19 (35%)	1 (4%)
Moderate (> 0 and < 19.9 units)	17 (31%)	5 (21%)
Absent	18 (33%)	18 (75%)

^a^High and moderate expression were defined as the boundary of the 75th percentile and obtained from the cumulative frequency of all expressing samples (see “Materials and methods”)

**Fig. 2:**
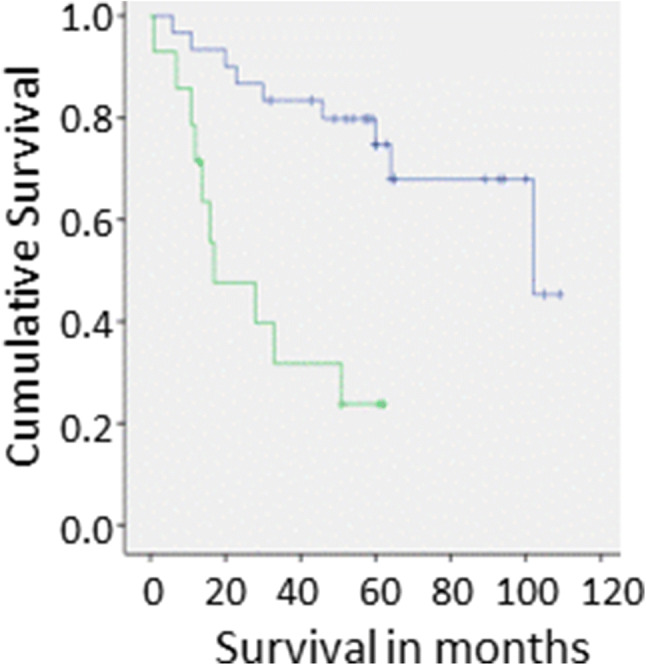
Kaplan–Meier analysis of overall survival in relation to levels of iRhom2 protein expression determined on western blots. Overexpression (green line: lower line) and normal expression (blue line: upper line) were defined using cumulative frequency (see “Materials and methods”). Censored data (i.e. living patients whose follow-up period is less than 120 months) is represented as a vertical bar on the relevant line. Four samples had missing survival data. (Color figure online)

No significant correlations were observed between iRhom2 protein expression in tumours and their clinicopathological features, except that high levels of iRhom2 expression were shown to correlate negatively with overall survival (P < 0.0005) (Fig. 2).

### Impact of altered expression of iRhom2 in cell lines

In contrast to data in the literature, the rate of proliferation was not increased in any of the cell lines by overexpression of iRhom2 (Supplementary Figs. 1 & 2), but was reduced in Liv37K and, to a lesser degree, in NOK-hTERT, while having no apparent effect on PE/CA-PJ15.

**Fig. 3:**
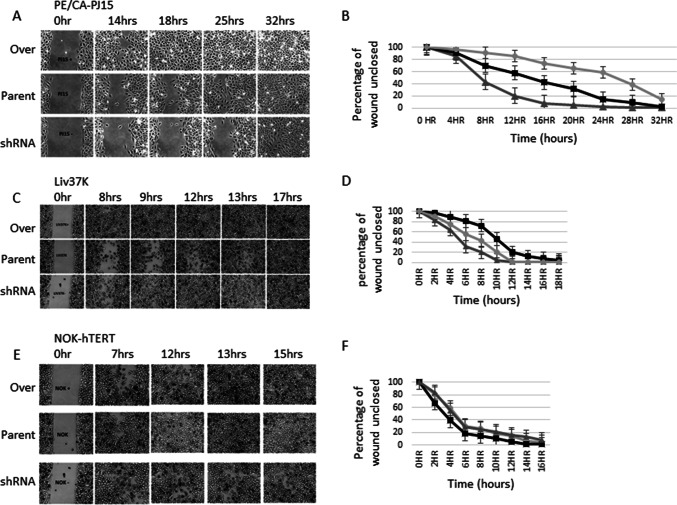
2D migration of oral cell lines. **a**, **c**, **e** Freeze frame photography at the time points indicated. **b**, **d**, **f** Graphical representations of the rates of wound closure showing the amount of scratch remaining unclosed at the time points indicated. **a**, **b** PE/CA-PJ15 cell line variants; **c**, **d** Liv37K variants; **e**, **f** NOK-hTERT variants. Black line with squares: wild type (WT); grey line with triangles: overexpressing clone; pale line with circles: shRNA knockdown clone

Despite the lack of increase in cell proliferation, PE/CA-PJ15 and Liv37K clones with up-regulated iRhom2 migrated approximately twice as fast as their respective wild-type variants (Figs 3a–d), with the knockdown clone of PE/CA-PJ15 showing a slower rate of migration than the wild type variant (Fig. 3a, b). Neither iRhom2 over-expression nor shRNA knock-down appeared to alter the rate of migration of NOK-hTERT cells (Fig. 3e, f).

## Discussion

A role for iRhom2 has been proposed in oesophageal and ovarian cancer [4], but evidence for a specific role in HNSCC has not been described. We have now shown that iRhom2 protein expression is often upregulated in OSCC in comparison to adjacent normal tissues (P < 0.05). Furthermore, increased iRhom2 expression correlated with poor survival (P < 0.0005), but did not correlate with any other clinicopathological indicators of prognosis, such as extracapsular spread or tumour size, suggesting that it may be an independent prognostic indicator. However, the sample size was too small to assess this statistically.

iRhom2 has been shown to be involved in diverse signalling events such as the trafficking/activation of important growth and signalling factors, e.g. ADAM17, EGFR, and TGFα (transforming growth factor α) [2, 3]. ADAM17, also known as TACE (Tumour Necrosis Factor-α converting enzyme), is involved in the shedding of signalling molecules such as TNFα, TGFα, EGF, Notch1 and CD44, and adhesion molecules such as L-selectin, syndecans, CAMs (Cell Adhesion Molecules) and cadherins [11–13]. Following its synthesis, ADAM17 protein is thought to be largely stored in the ER in immature form, prior to maturation in the Golgi apparatus and onward trafficking to the cell membrane [14]. Therefore, only a fraction of the mature / activated form is demonstrated to be available at the cell membrane at any one time [14] and trafficking to the cell membrane is thought to be orchestrated by iRhom2 [2]. Increased ADAM17 expression has been shown to lead to poor survival in OSCC orthotopic animal models [15] and may thus be implicated as the downstream initiator of iRhom2.

To augment our observational study on clinical samples, we both over-expressed and knocked down iRhom2 expression in two oral cancer cell lines and one immortalised normal oral keratinocyte cell line. Interestingly, cell proliferation was not shown to be augmented by increased expression of iRhom2 and these data are in contrast to the literature that suggests a proliferative role for iRhom2 [16, 17]. Upregulation of iRhom2 in the two cancer cell lines increased their rate of 2D migration in a wound healing assay but did not confer a similar advantage to a non-cancerous oral epithelial cell line. This data again implicates ADAM17 as the effector as this has been associated with poor prognosis and promotion of oral cancer cell line migration via inactivating cleavage of CXCL1, a cell adhesion regulator [18]. Furthermore, motility of cancer associated fibroblasts in culture has been shown to be dependent on iRhom2-dependent, ADAM17-mediated cleavage of TGF beta receptor 1 [19].

These data implicate activation of the iRhom2-ADAM17 pathway in the pathogenesis of OSCC, and suggest that this system works by influencing or increasing rate of cell migration. Deregulation of ADAM17 has previously been implicated in head and neck squamous cell carcinoma (HNSCC) [20], with up-regulation of ADAM17, as well as increased sheddase activity, correlating with tumour aggressiveness, rate of growth and prognosis in this and other cancers [20–23]. Increased ADAM17 sheddase activity has also been demonstrated following increased expression of the protein in head and neck cancer cell lines [20]. Although the exact mechanism remains largely unclear, there is some evidence to suggest that upregulated ADAM17 in head and neck cancer plays key roles in tumour initiation and progression via proteolytic and/or adhesive properties [20]. This may relate in turn to our data indicating poor survival in those patients whose cancers overexpressed iRhom2. However, additional research is required to completely unravel the signalling pathways involved and confirm whether modulation of iRhom2 expression may be a future target for therapy or useful as a prognostic marker.

## Electronic supplementary material

Below is the link to the electronic supplementary material.

Supplementary file1 (GIF 80 kb)

Supplementary file2 (GIF 13 kb)

Supplementary file3 (DOCX 12 kb)
