# Supplementary figures and images for: iRhom2 in the pathogenesis of oral squamous cell carcinoma
Source: Mol Biol Rep. 2020 Mar 31;47(5):3987–92. doi: 10.1007/s11033-020-05381-y (PMC7239832; doi:10.1007/s11033-020-05381-y)

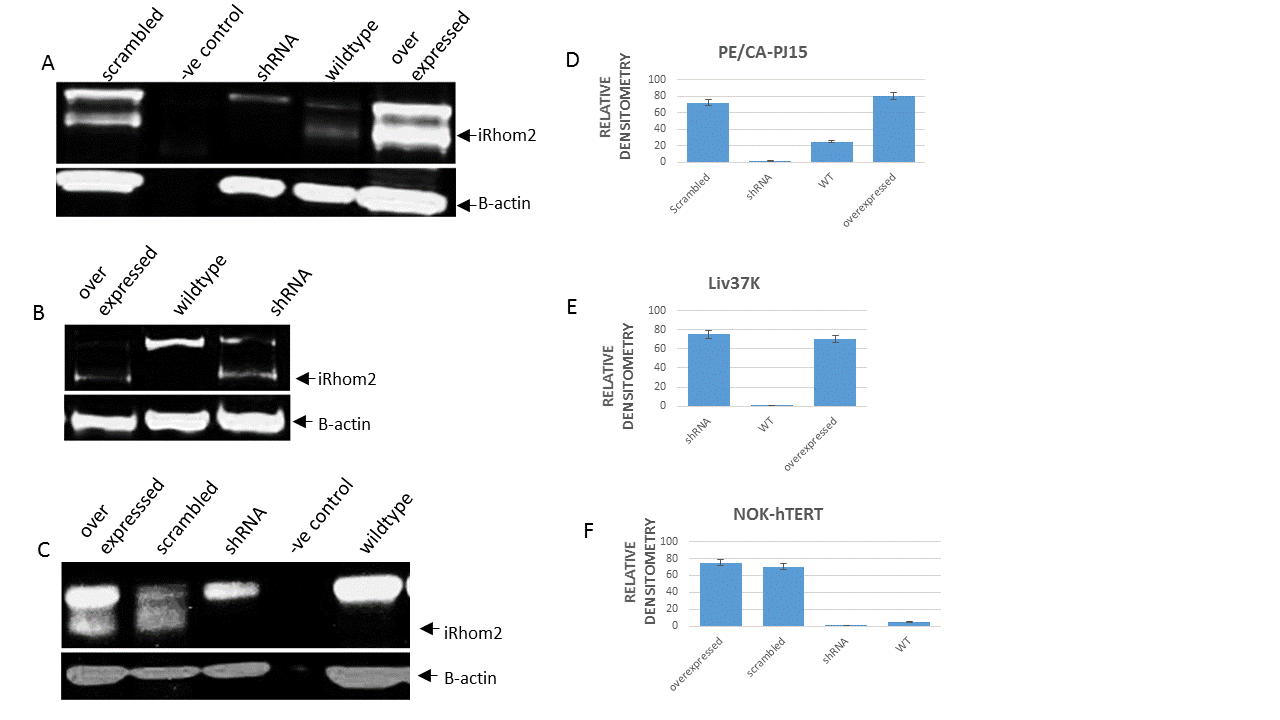

Supplement: Supplementary file 1 — Supplementary file1 (GIF 80 kb) [file 11033_2020_5381_MOESM1_ESM.gif]

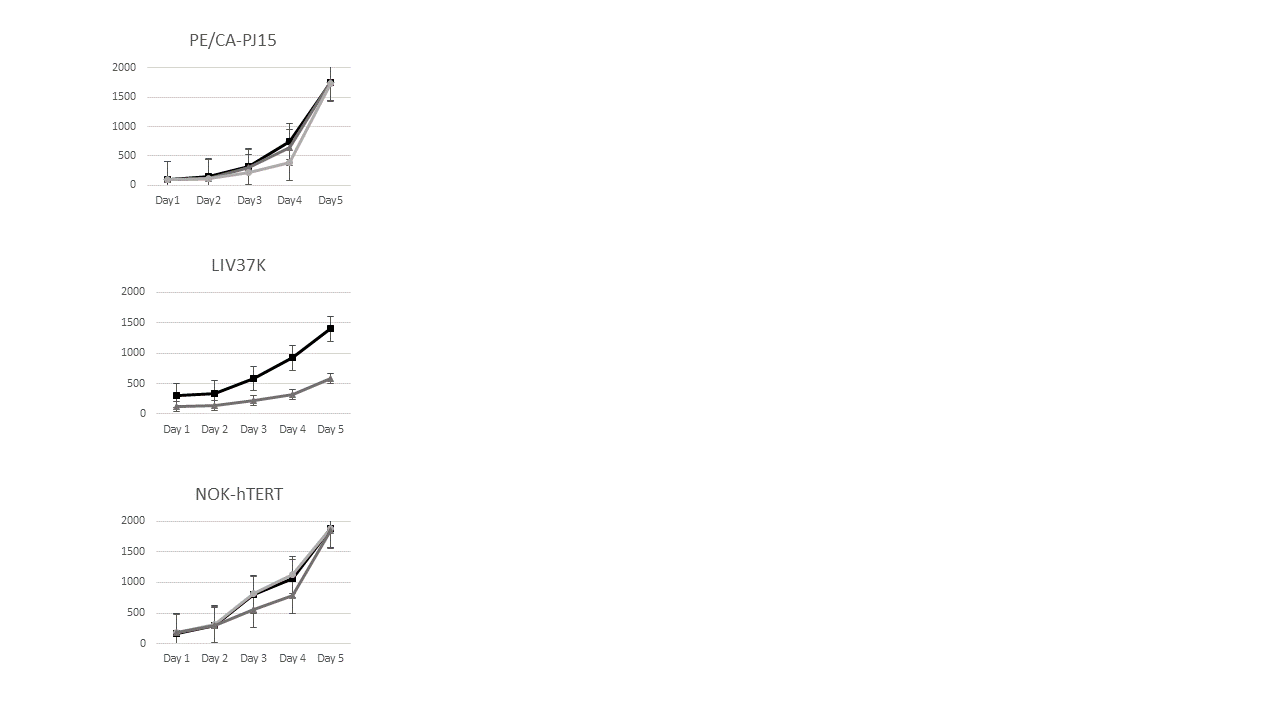

Supplement: Supplementary file 2 — Supplementary file2 (GIF 13 kb) [file 11033_2020_5381_MOESM2_ESM.gif]
