# Supplementary material for: iRhom2 in the pathogenesis of oral squamous cell carcinoma
Source: Mol Biol Rep. 2020 Mar 31;47(5):3987–92. doi: 10.1007/s11033-020-05381-y (PMC7239832; doi:10.1007/s11033-020-05381-y)
Supplement: Supplementary file 3 — Supplementary file3 (DOCX 12 kb) [file 11033_2020_5381_MOESM3_ESM.docx]

**Supplementary Fig. 1** Overexpression and shRNA knockdown of iRhom2 isoform 2 in oral cell lines**.** Western blot (A, B, C), and protein quantification (D, E, F) of iRhom2 using densitometry (arbitrary units). A & D: PE/CA-PJ15; B & E: Liv37K; C & F: NOK-hTERT. WT: wild type; Over: overexpressing clone; shRNA: shRNA knockdown of the overexpressing clone; scrambled: shRNA control. Arrows on western blots indicate the expected position of iRhom2 isoform 2 and beta actin. Negative control contains no protein and is not analysed in the quantification charts.

**Supplementary Fig. 2** Crystal violet proliferation assay. Viable cell population, represented by the absorbance, is plotted against time in days. Black line with squares: wild type (WT); grey line with triangles: overexpressing clone; pale line with circles: shRNA knockdown clone. It was not possible to obtain a Liv37K knockdown for comparison, despite using 2 shRNA molecules separately and in combination
